# Systemic Lupus Erythematosus with Refractory Immune Thrombocytopenia Progressing to Catastrophic Anti-Phospholipid Syndrome During Thrombopoietin Receptor Agonist Therapy: A Case Report

**DOI:** 10.3390/jcm14093091

**Published:** 2025-04-29

**Authors:** Sang Wan Chung, You-Jung Ha

**Affiliations:** 1 Division of Rheumatology, Department of Internal Medicine, Kyung Hee University College of Medicine, Kyung Hee University Hospital, Seoul 02447, Republic of Korea; wanyworld83@gmail.com; 2 Division of Rheumatology, Department of Internal Medicine, Seoul National University Bundang Hospital, Seongnam 13620, Republic of Korea; 3 Department of Internal Medicine, Seoul University College of Medicine, Seoul 03080, Republic of Korea

**Keywords:** immune thrombocytopenia, systemic lupus erythematosus, romiplostim

## Abstract

**Background/Objectives**: Autoimmune thrombocytopenia is a common manifestation of systemic lupus erythematosus (SLE). Its main treatments are glucocorticoids, intravenous immunoglobulin, and immunosuppressants, but thrombopoietin mimetics may be considered with refractory to conventional treatment. Romiplostim, a thrombopoietin receptor agonist, has been approved for increasing platelet counts in corticosteroid-refractory chronic immune thrombocytopenia. However, data on its long-term safety and efficacy in patients with SLE are still lacking. **Case Presentation**: We present the case of a 55-year-old woman with SLE and refractory immune thrombocytopenia who developed bilateral adrenal hemorrhage and progressed to fatal catastrophic anti-phospholipid syndrome while using romiplostim.

## 1. Introduction

Hematological abnormalities such as hemolytic anemia, leukopenia, thrombocytopenia, lymphadenopathy, and/or splenomegaly are common manifestations of systemic lupus erythematosus (SLE) at the time of diagnosis and throughout the course of the disease [1]. Thrombocytopenia in SLE is usually non-severe, but approximately 3–10% of patients experience severe thrombocytopenia with a platelet count below 20–30,000/mm^3^ or clinically significant bleeding [2,3]. Severe immune thrombocytopenia (ITP) in SLE warrants therapeutic management, consisting of corticosteroids with or without intravenous immunoglobulin (IVIg) in the acute stage and several immunosuppressants for chronic maintenance. However, a significant number of patients do not respond to the aforementioned treatment or relapse after it. Splenectomy or thrombopoietin receptor agonists may be considered in cases of recurrent or resistant disease [4].

Romiplostim is a thrombopoietin receptor agonist (TPO-RA) that has been approved as a second-line treatment in patients with chronic refractory ITP. Although there have been concerns about adverse events associated with romiplostim—such as bone marrow reticulin fibrosis, which may progress to myelofibrosis, as well as hematologic malignancies and thrombosis—previous studies have demonstrated no overrepresented risk of these adverse events in the treatment of ITP [5,6]. However, the safety data of TPO-RA for ITP related to SLE are limited. Recently, several case reports and retrospective data of thrombotic events in patients with SLE or anti-phospholipid syndrome (APS) treated with romiplostim for refractory ITP have been reported [7,8].

We report the case of a patient with SLE-associated ITP who developed bilateral adrenal hemorrhage and progressed to catastrophic APS (CAPS), leading to death during romiplostim therapy.

## 2. Case Report

A 55-year-old woman was diagnosed with SLE in 2011, based on proteinuria, hypocomplementemia, and positivity for antinuclear antibody, anti-dsDNA, lupus anticoagulant (LA), β2-GPI antibody, and anti-cardiolipin antibody. Four months before admission, severe immune thrombocytopenia (<5000/mm^3^) with petechiae and leukopenia developed without the involvement of other organs in SLE. The patient was treated with high-dose glucocorticoids, IVIg, azathioprine, danazol, rituximab (375 mg/m^2^/week for four weeks), and eltrombopag. However, the treatment response was inadequate, with a platelet count persistently below 5000/mm^3^. Two months prior to admission, romiplostim was started at a dose of 1 μg/kg/week and increased to a maximum of 8 μg/kg/week with combined treatment of hydroxychloroquine, danazol, and prednisone. The patient achieved a partial response (platelet count between 30,000/mm^3^ and 50,000/mm^3^) after reaching the maximum dose of romiplostim. After that, since her proteinuria was aggravated (>1 g/day), mycophenolate mofetil (1000 mg/day) was added, but MMF and danazol were discontinued due to newly developed hepatitis of unknown causes. Her platelet count remained above 30,000/mm^3^ for over one month under the maintenance dose of prednisolone (10 mg/day) and romiplostim 8 μg/kg/week.

**Figure 1:**
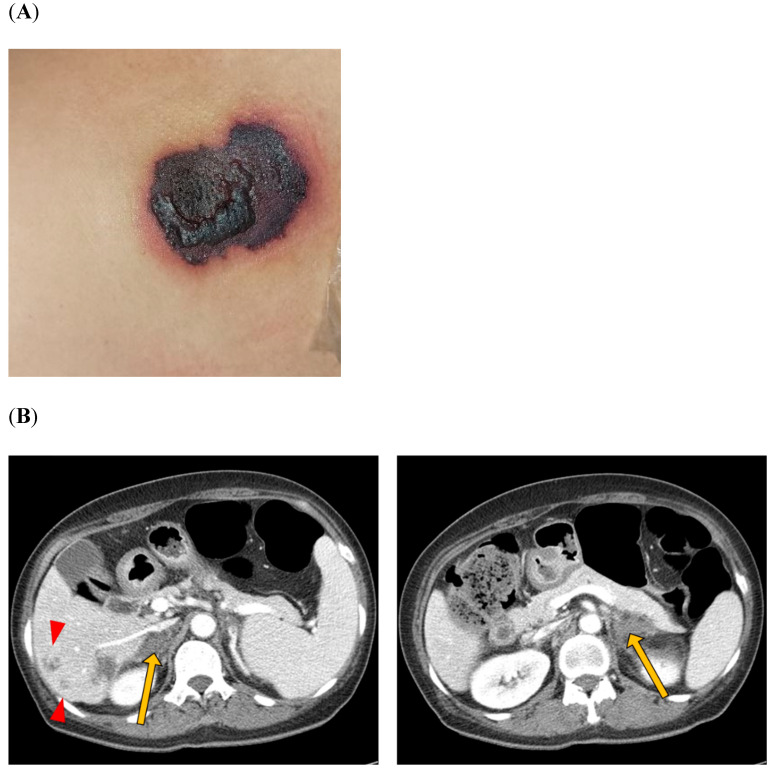
(**A**) Hemorrhagic bullae with erythema located on the abdomen. (**B**) An abdominal computed tomography scan showing multiple low attenuating lesions in the bilateral adrenal glands (yellow arrows) and low attenuating lesions in the liver (red arrowheads).

**Figure 2:**
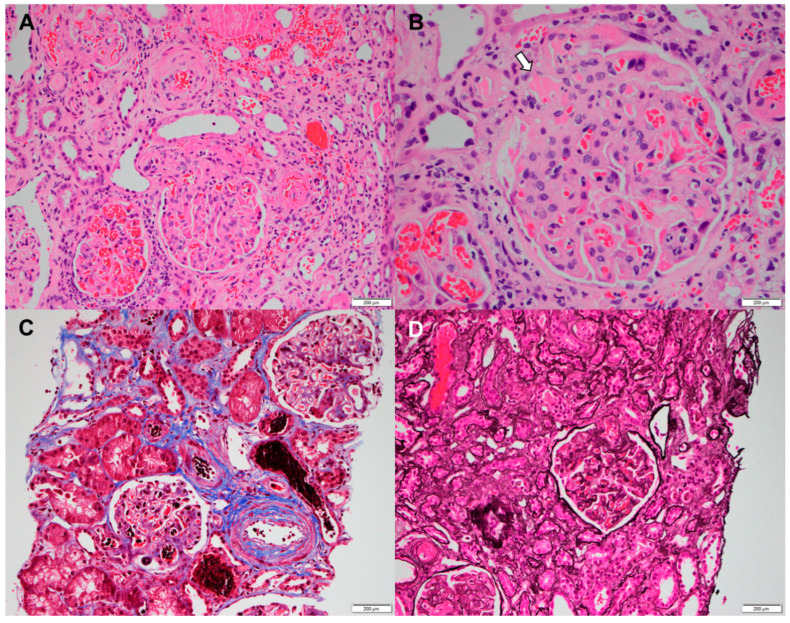
Histopathologic features of a kidney biopsy. (**A**) Fibrin thrombi in the glomerular capillary lumen and arteriole and vascular endothelial swelling with fibrinoid necrosis (hematoxylin and eosin staining ×200). (**B**) Fibrin thrombi at the vascular pole (white arrow, H & E staining, ×400). (**C**) Thrombi in the glomerular capillaries and arterioles (Masson’s trichrome stain ×200). (**D**) Glomerular capillary loops and arterioles containing abundant erythrocytes (periodic acid silver-methenamine stain ×200).

**Figure 3:**
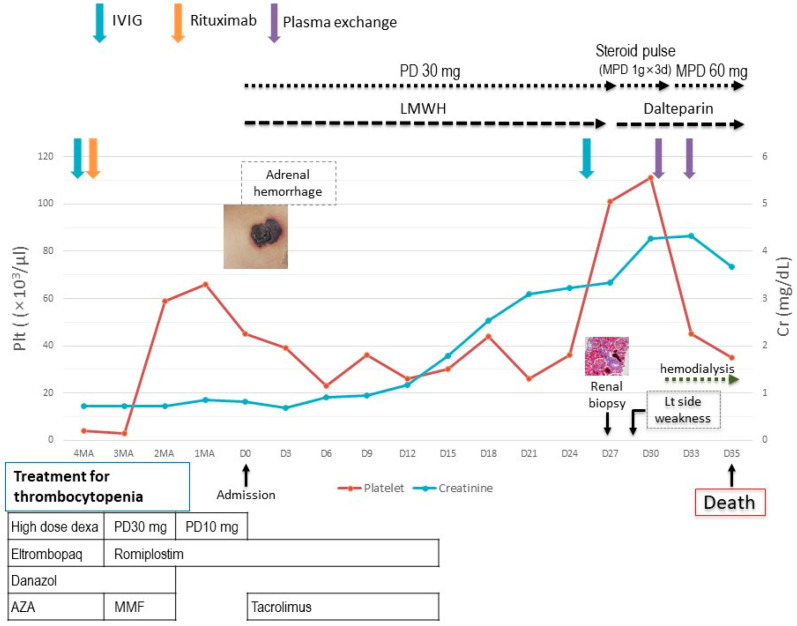
Timeline chart illustrating the patient’s medication history, major clinical events, and corresponding changes in platelet count and renal function before and after hospitalization. IVIG, intravenous immunoglobulin; PD, prednisolone; MPD, methylprednisolone; LMWH, low molecular weight heparin; Plt, Platelet; Cr, creatinine; MA, month ago; D, day; dexa, dexamethasone; AZA, azathioprine; and MMF, mycophenolate mofetil.

Two months later, the patient visited the emergency room because of severe bilateral flank pain accompanied by hemorrhagic bullae located on the abdomen (Figure 1). Laboratory findings showed white blood cells of 9400/mm^3^ (reference range [RR] 4000–10,000/mm^3^), hemoglobin of 13.5 g/dL (RR 12–16 g/dL), and a platelet count of 45,000/mm^3^ (RR 130,000–400,000/mm^3^). Prothrombin time international normalized ratio (PT INR) was 0.99 (RR 0.9–1.2), and activated partial thromboplastin time (aPTT) was markedly prolonged at 113.9 sec (RR 29–43 s). D-dimer was elevated to 6.2 µg/mL (RR < 0.50 µg/mL), and serum creatinine (SCr) was normal (0.69 mg/dL; RR 0.7–1.4 mg/dL). Haptoglobin and total bilirubin levels were within the normal ranges. Fibrinogen was higher than 1000 mg/dL (RR 200–400 mg/dL), and lactate dehydrogenase (LDH) was mildly elevated 434 IU/L (RR 100–225 IU/L). Anti-neutrophil cytoplasmic antibodies (ANCAs) were negative, and complement C4 was mildly decreased to 6.75 mg/dL (RR ≥ 10 mg/dL). Her LA was positive, and anti-β2-glycoprotein I immunoglobulin G was positive at a high titer (51.0 GPL unit; RR ≤ 20 GPL). Peripheral blood smear revealed the normochromic normocytic red blood cells with mild rouleaux formation and a marked decreased platelet number, but no schistocytes were observed. Abdominal computed tomography revealed multiple low attenuating lesions in the bilateral adrenal glands, representing adrenal hemorrhage and low-attenuation lesions in the liver (Figure 1). It was thought to be a manifestation of APS requiring anticoagulation. However, the patient’s platelet count was still low (20,000–40,000/mm^3^), so we started a half dose of subcutaneous low molecular weight heparin (1 mg/kg). Prednisolone was increased to 30 mg/day, and tacrolimus was added for proteinuria. On hospital day 15, acute kidney injury (SCr 1.35 mg/dL) was developed. Despite hydration and the discontinuation of possible nephrotoxic drugs such as tacrolimus, azotemia was aggravated (SCr 3.33 mg/dL on hospital day 25). Urinalysis showed 3+ in red blood cells, and proteinuria was approximately 1 g/day. For the differential diagnosis of renal dysfunction, a renal biopsy was performed following the administration of IVIg on day 26 of hospitalization. The histopathological findings of the kidney were compatible with thrombotic microangiopathy (Figure 2), and romiplostim was discontinued. On day 28 of hospitalization, the patient presented with sudden-onset left-sided weakness. Magnetic resonance imaging of the brain showed multifocal infarction in the right frontal and left parietal lobes, suggesting embolic infarction. In addition, innominate vein thrombosis was detected on the upper extremity ultrasonography performed for arm edema. Echocardiography revealed a decreased left ventricle (LV) ejection fraction (26%) with mid-to-apical LV akinesia, suggesting ischemic cardiomyopathy. Anticoagulation was switched to subcutaneous dalteparin at a full therapeutic dose. The diagnosis of CAPS was made based on the involvement of three major organs (kidney, brain, and heart) within one week. The patient was treated with intravenous methylprednisolone pulse (1 g/day for three days) and plasma exchange. Her subsequent hospital course was complicated by end-stage renal disease, and hemodialysis was required. Ultimately, she died of cardiac arrest on day 32 of hospitalization (Figure 3).

## 3. Discussion

ITP in SLE is associated with an unfavorable prognosis and higher mortality [3]. The pathogenesis of thrombocytopenia in SLE is heterogeneous, but the most common mechanism is believed to be an increased platelet clearance and impaired platelet production mediated by antiplatelet autoantibodies. Two types of autoantibodies have been described as follows: anti-GPIIb/IIIa and anti-TPO receptors [9]. High-dose glucocorticoids are the cornerstone of the initial treatment for SLE-ITP. Second-line agents include hydroxychloroquine, danazol, and immunosuppressive drugs such as azathioprine, cyclosporine, mycophenolate mofetil, cyclophosphamide, and biological therapies such as rituximab. TPO-RAs, romiplostim, and eltrombopag have been recently used in the treatment of ITP, and they also seem to play a role in SLE-ITP [10].

APS is a multisystem autoimmune thrombotic condition characterized by the occurrence of arterial and venous thrombosis and/or pregnancy-related morbidities in the presence of anti-phospholipid antibodies (aPL). Bilateral adrenal hemorrhage is a rare manifestation of APS and may present during the course of CAPS in some patients [11]. The cornerstone of treatment for CAPS is therapeutic anticoagulation and corticosteroids. Therapeutic plasma exchange is an established intervention, particularly for CAPS patients with microangiopathic features or renal involvement. Immunosuppressive agents such as cyclophosphamide and rituximab may be an additional effective therapy in SLE-associated CAPS [12,13].

Our patient started anticoagulation therapy after the occurrence of bilateral adrenal hemorrhage. However, the persistent low platelet count and former treatment failure with most drugs for ITP limited the use of anticoagulants at a full therapeutic dose and the discontinuation of romiplostim.

TPO-RAs are generally well tolerated, and the most frequently reported side effects are headache, nausea, diarrhea, arthralgia, and nasopharyngitis. TPO-RA may be intrinsically related to thrombotic risk through platelet activation/aggregation [14]. However, data from a number of long-term prospective clinical trials have shown no significant increase in thrombotic adverse events among patients with chronic ITP [6]. However, the benefits of TPO-RA in SLE- or APS-ITP require further investigation. In 2018, a multicenter retrospective study by Guitton et al. reported the safety of TPO-RA in 18 patients with SLE-ITP [7]. In their study, 5 out of 18 patients experienced thrombotic events, and one of those treated with eltrombopag presented with CAPS. Four patients with serious unexpected thrombosis had APS or aPL. Prior to this study, only three cases of thrombotic adverse events during TPO-RA therapy in patients with SLE have been sporadically reported. Tomov et al. described a young female SLE patient without aPL presenting with renal thrombotic microangiopathy after romiplostim treatment [15]. In another case series, CAPS occurred in a 14-year-old female patient with aPL who received romiplostim [8]. The outcome of these patients was favorable with romiplostim cessation, which is different from our case. Because the presence of aPL itself increases the risk of thrombotic events, a comparative prospective study in a large-scale aPL-positive population is needed to confirm that the use of TPO-RA actually increases the thromboembolic risk synergistically. However, several cases of CAPS in SLE-ITP patients with aPL exposed to TPO-RA, including our fatal case, indicate that TPO-RA should be used with caution in such patients.

In conclusion, TPO-RAs may be a treatment option for refractory SLE-ITP, but the coexistence of APS or aPL should be screened before its use and taken into account when making treatment decisions.

## Data Availability

No new data were created or analyzed in this study. Data sharing is not applicable to this article.
